# Supplementary material for: Neural drive and motor unit characteristics after anterior cruciate ligament reconstruction: implications for quadriceps weakness
Source: PeerJ. 2023 Oct 6;11:e16261. doi: 10.7717/peerj.16261 (PMC10561646; doi:10.7717/peerj.16261)
Supplement: Supplemental Information 1 — Regression lines showing the MFR-RT and MUAPAMP-RT relationship are plotted using the y-intercept and slope group means from participant specific regression line and extend the mean length of regression line. Shaded area is standard error of measurement of the slope. Scatter plots depict the regression line slope and length of identified MU in each group and limb. Black bars depict the mean. Statistical comparisons are shown is p < 0.05 with Bonferroni correction. Solid brackets with asterisks indicate difference across Group x Limb. Brackets with feet indicate differences between Groups. [file peerj-11-16261-s001.pdf]

Slide 1

Quadriceps weakness after anterior cruciate ligament reconstruction: inability to recruit high-threshold motor units

Supplement 1

Results from 30% and 50% MVIC trials.

Slide 2

Torque Tracing

30%

50%

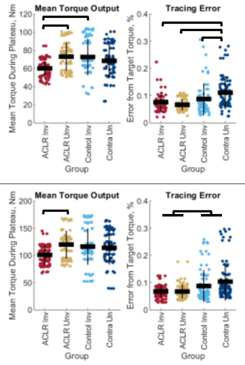

Mean torque output and force tracing performance during the 30% and 50% trials. Individual circles depict the predicted value from the mixed effect model. Solid horizontal bars represent the least squared means and vertical error bars are the standard deviation. *Post hoc* comparisons that reached statistical significance are denoted with brackets. Group effects are denoted with the footed bracket. Abbreviations: ACLR, anterior cruciate ligament reconstruction; MVIC, maximum voluntary isometric contraction; Nm, Newton-meter.

Slide 3

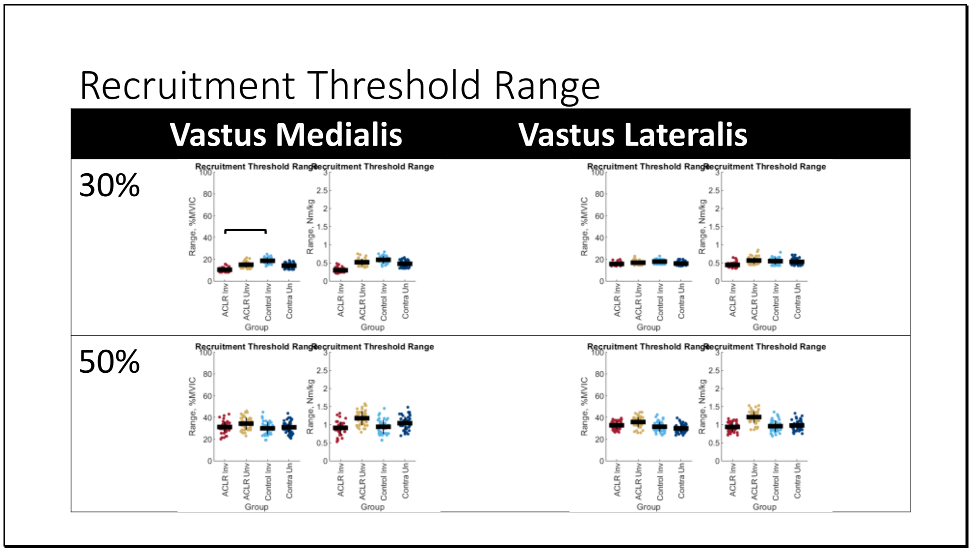

Relative and mass-normalized recruitment threshold range for MUs identified in the vastus medialis and vastus lateralis during the 30% and 50% trials. Individual circles depict the predicted value from the mixed effect model. Solid horizontal bars represent the least squared means and vertical error bars are the standard deviation. *Post hoc* comparisons that reached statistical significance are denoted with brackets. Abbreviations: ACLR, anterior cruciate ligament reconstruction; MVIC, maximum voluntary isometric contraction; Nm/kg, Newton-meter per kilogram.

Slide 4

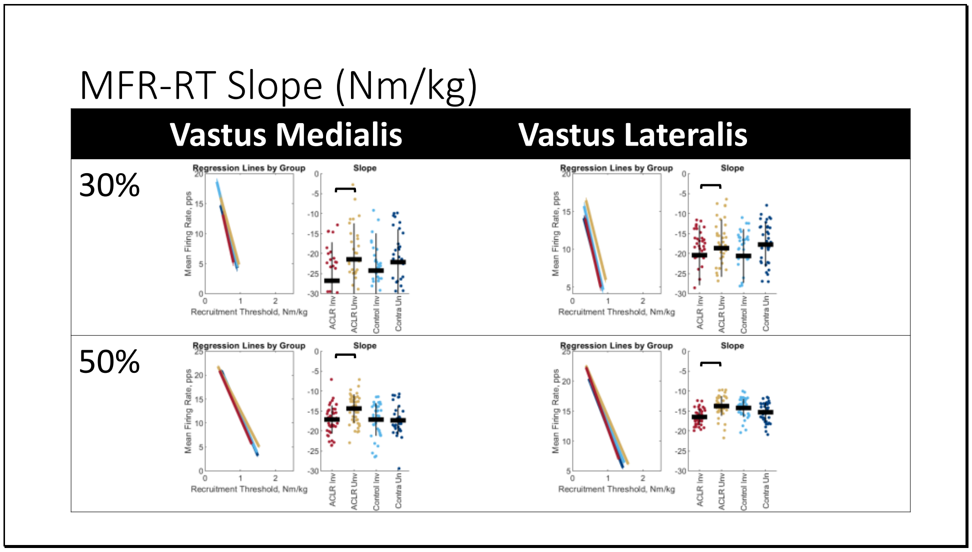

Mean firing rate to mass-normalized recruitment threshold slope comparisons for MUs identified in the vastus medialis and vastus lateralis during the 30% and 50% trials. In the left plots, dashed lines represent the mean firing rate to recruitment threshold relationship across the group*limb levels. Lines are plotted with average slopes and mass-normalized recruitment threshold ranges without controlling for y-intercept. In the right plots, individual circles depict the predicted value from the mixed effect model. Solid horizontal bars represent the least squared means and vertical error bars are the standard deviation. *Post hoc* comparisons that reached statistical significance are denoted with brackets. Abbreviations: ACLR, anterior cruciate ligament reconstruction; MVIC, maximum voluntary isometric contraction; Nm/kg, Newton-meter per kilogram; pps, pulses per second.

Slide 5

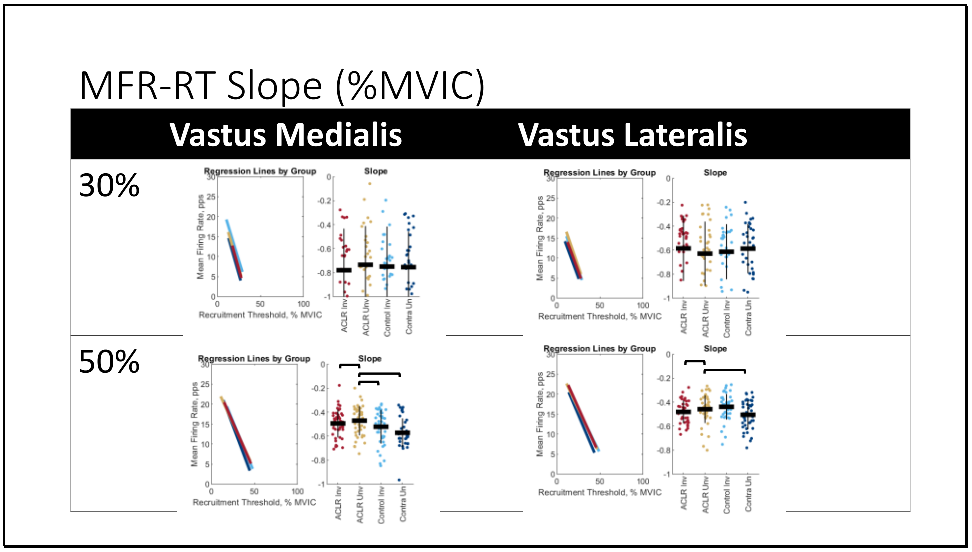

Mean firing rate to relative recruitment threshold slope comparisons for MUs identified in the vastus medialis and vastus lateralis during the 30% and 50% trials. In the left plots, dashed lines represent the mean firing rate to recruitment threshold relationship across the group*limb levels. Lines are plotted with average slopes and relative recruitment threshold ranges without controlling for y-intercept. In the right plots, individual circles depict the predicted value from the mixed effect model. Solid horizontal bars represent the least squared means and vertical error bars are the standard deviation. *Post hoc* comparisons that reached statistical significance are denoted with brackets. Abbreviations: ACLR, anterior cruciate ligament reconstruction; MVIC, maximum voluntary isometric contraction; Nm/kg, Newton-meter per kilogram; pps, pulses per second.

Slide 6

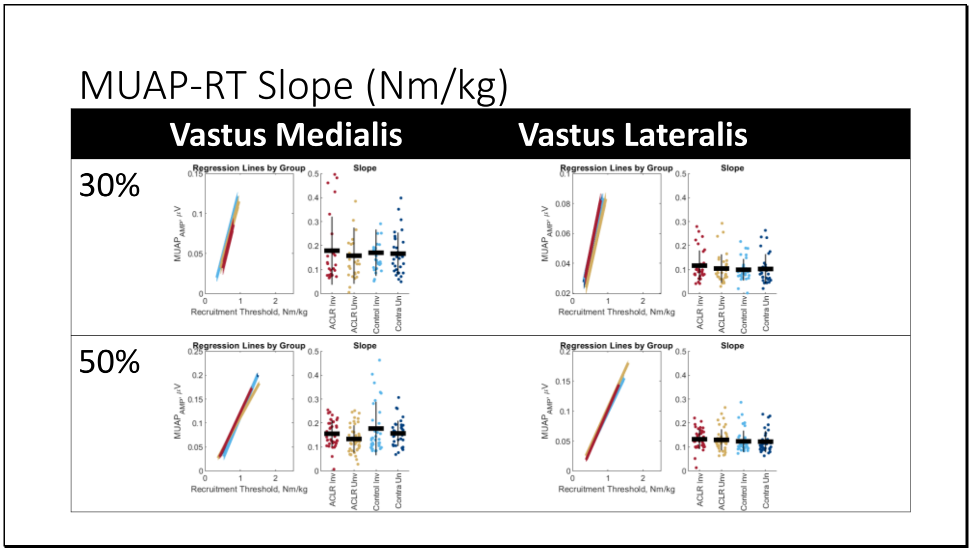

Motor unit action potential amplitude to mass-normalized recruitment threshold slope comparisons for MUs identified in the vastus medialis and vastus lateralis during the 30% and 50% trials. In the left plots, dashed lines represent the action potential amplitude to recruitment threshold relationship across the group*limb levels. Lines are plotted with average slopes and mass-normalized recruitment threshold ranges without controlling for y-intercept. In the right plots, individual circles depict the predicted value from the mixed effect model. Solid horizontal bars represent the least squared means and vertical error bars are the standard deviation. *Post hoc* comparisons that reached statistical significance are denoted with brackets. Abbreviations: ACLR, anterior cruciate ligament reconstruction; MVIC, maximum voluntary isometric contraction; Nm/kg, Newton-meter per kilogram; uV, microvolts.

Slide 7

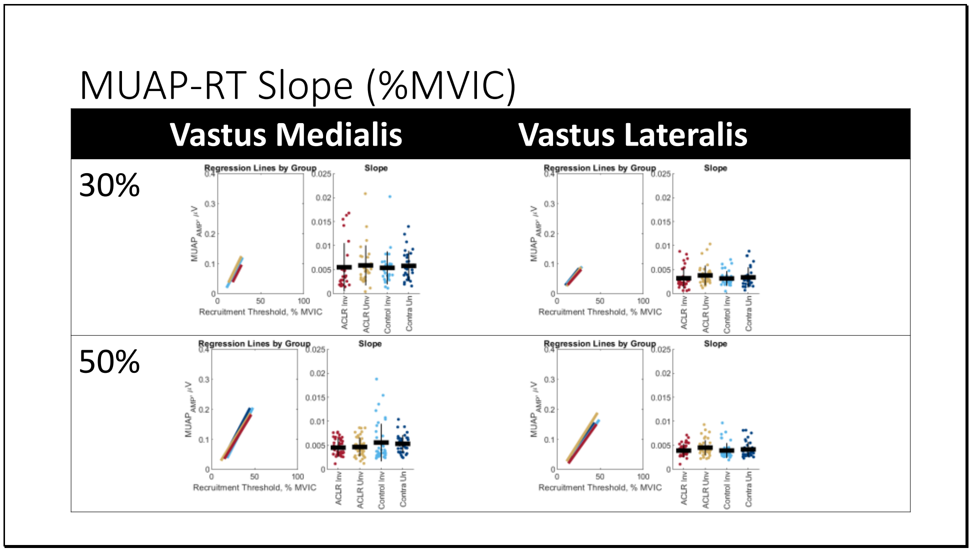

Motor unit action potential amplitude to relative recruitment threshold slope comparisons for MUs identified in the vastus medialis and vastus lateralis during the 30% and 50% trials. In the left plots, dashed lines represent the action potential amplitude to recruitment threshold relationship across the group*limb levels. Lines are plotted with average slopes and relative recruitment threshold ranges without controlling for y-intercept. In the right plots, individual circles depict the predicted value from the mixed effect model. Solid horizontal bars represent the least squared means and vertical error bars are the standard deviation. *Post hoc* comparisons that reached statistical significance are denoted with brackets. Abbreviations: ACLR, anterior cruciate ligament reconstruction; MVIC, maximum voluntary isometric contraction; Nm/kg, Newton-meter per kilogram; pps, pulses per second.
